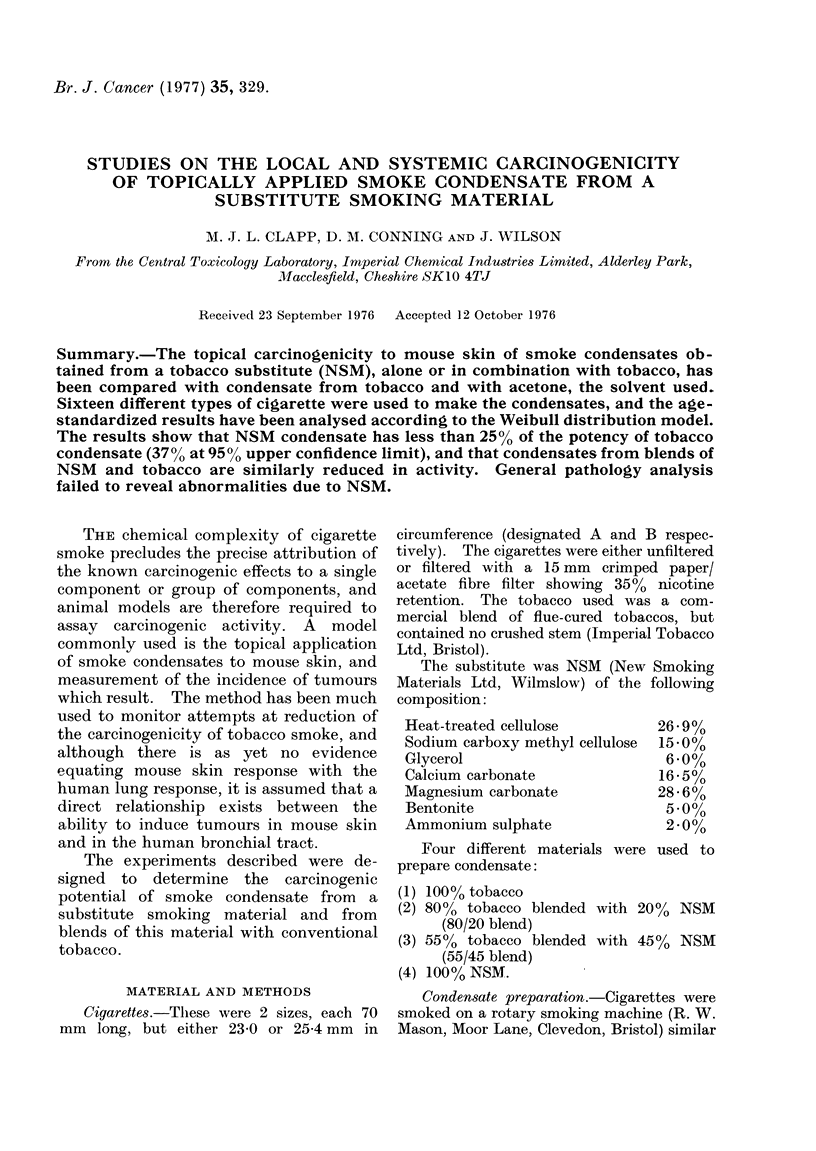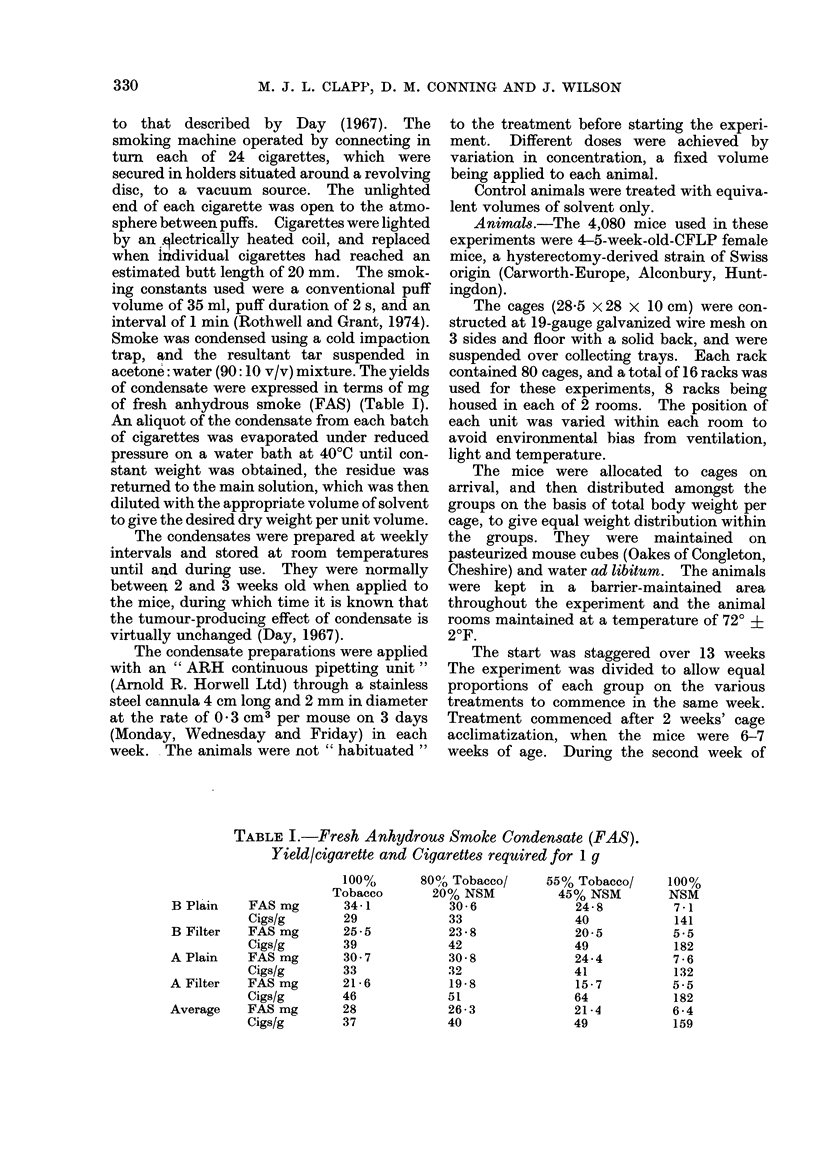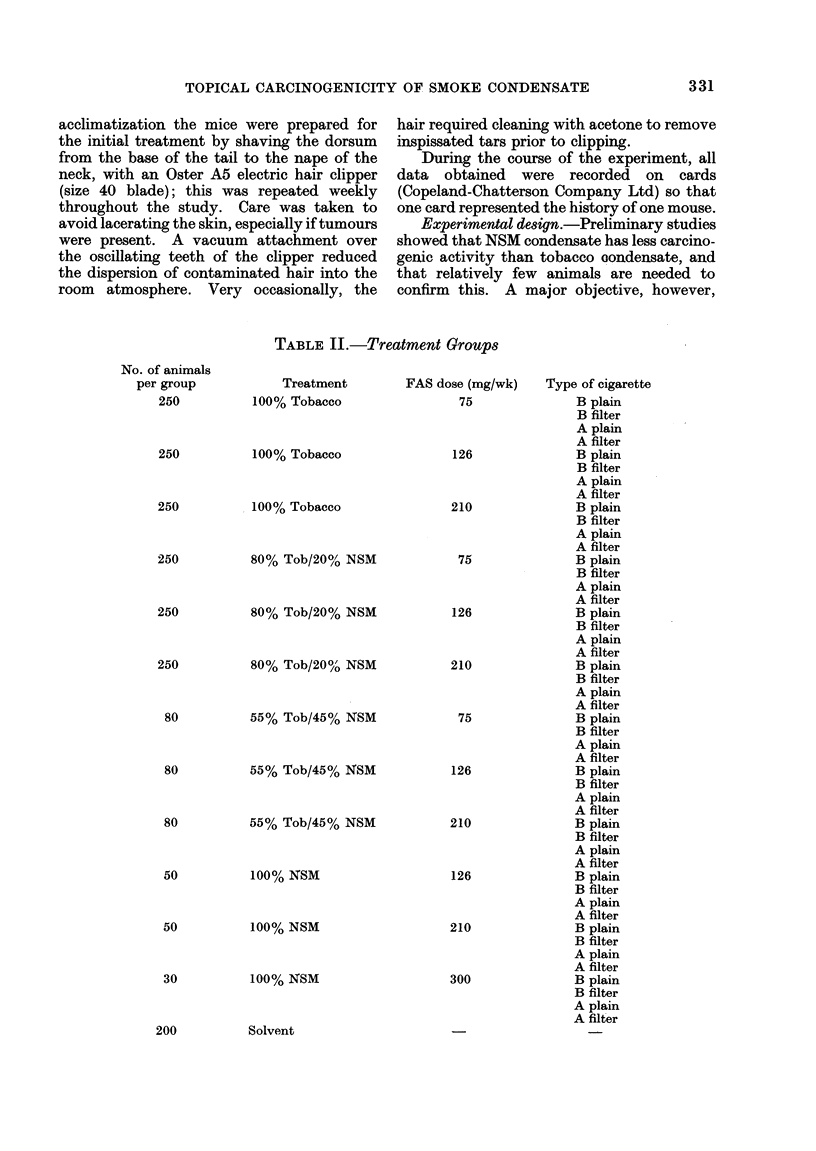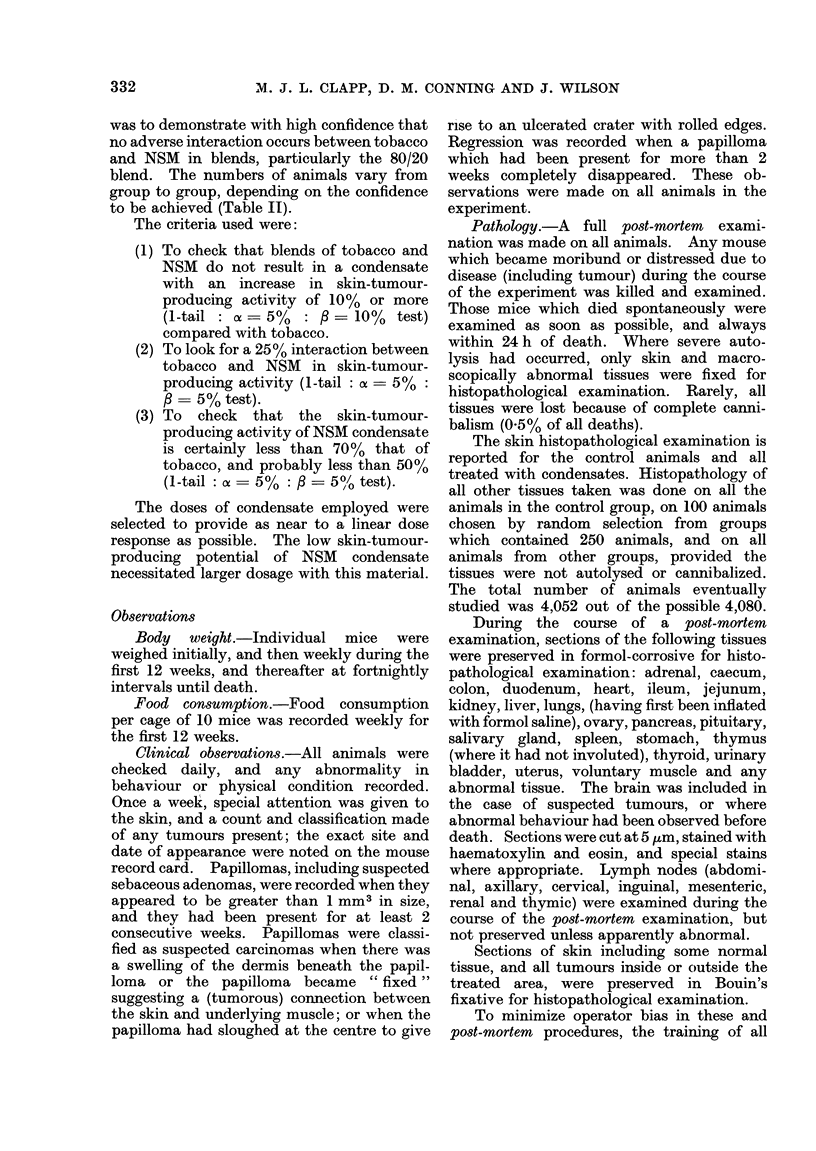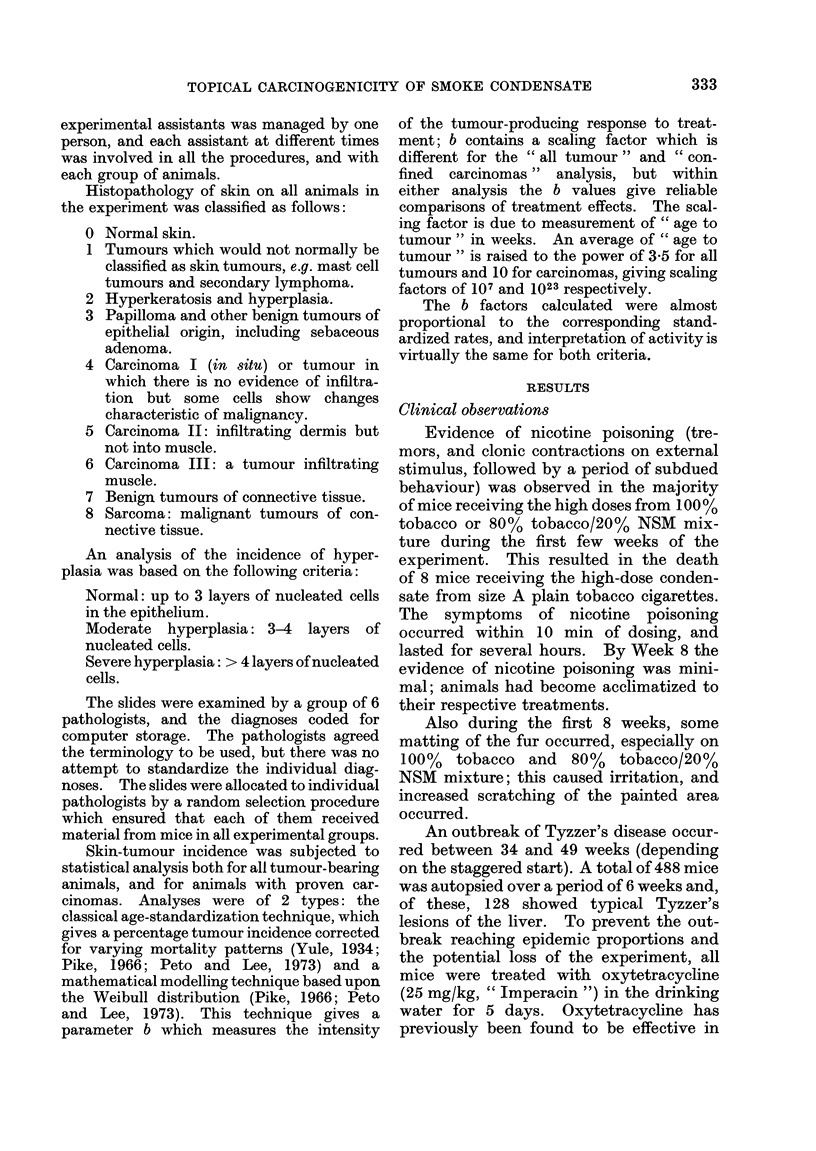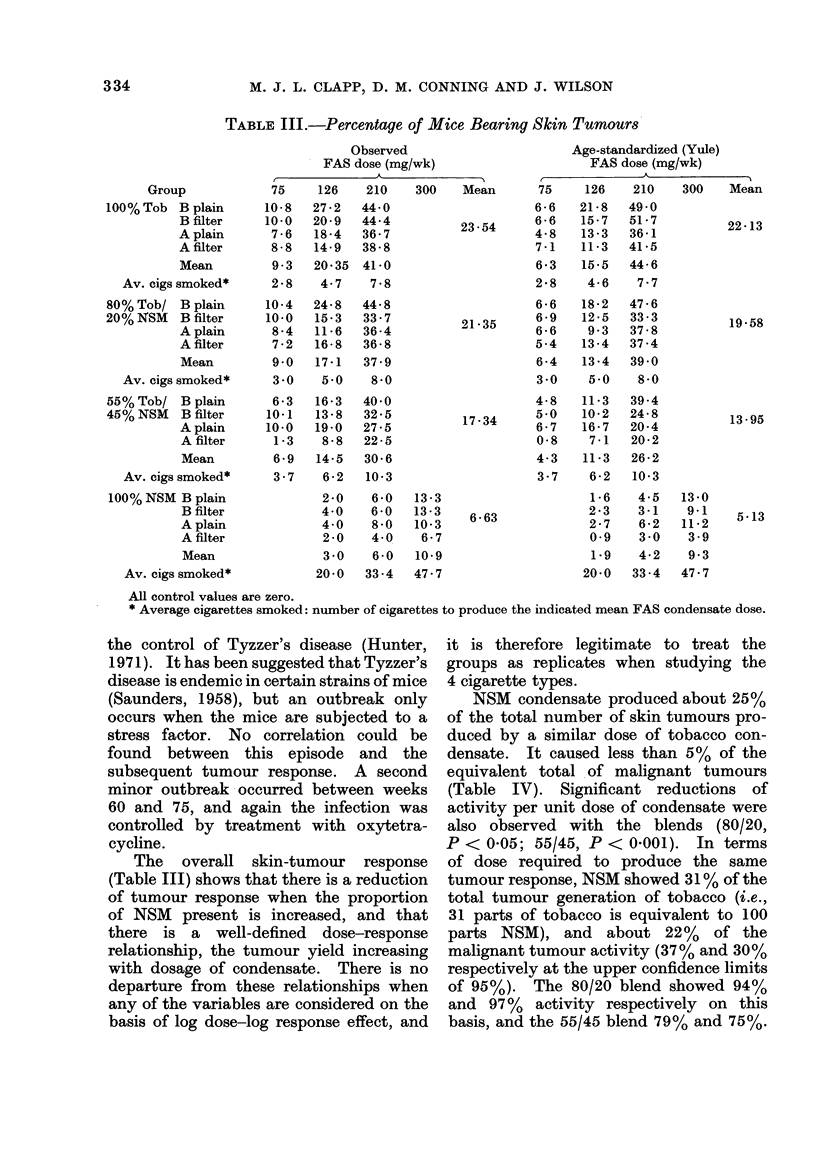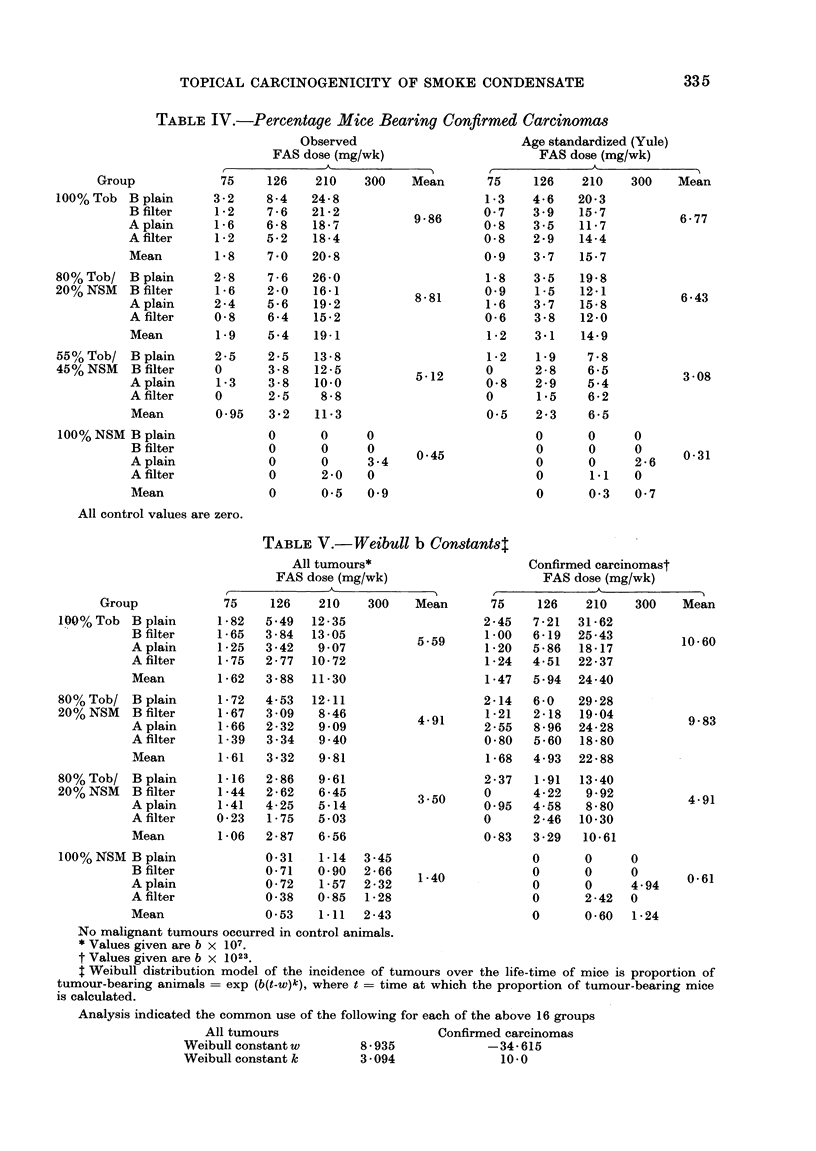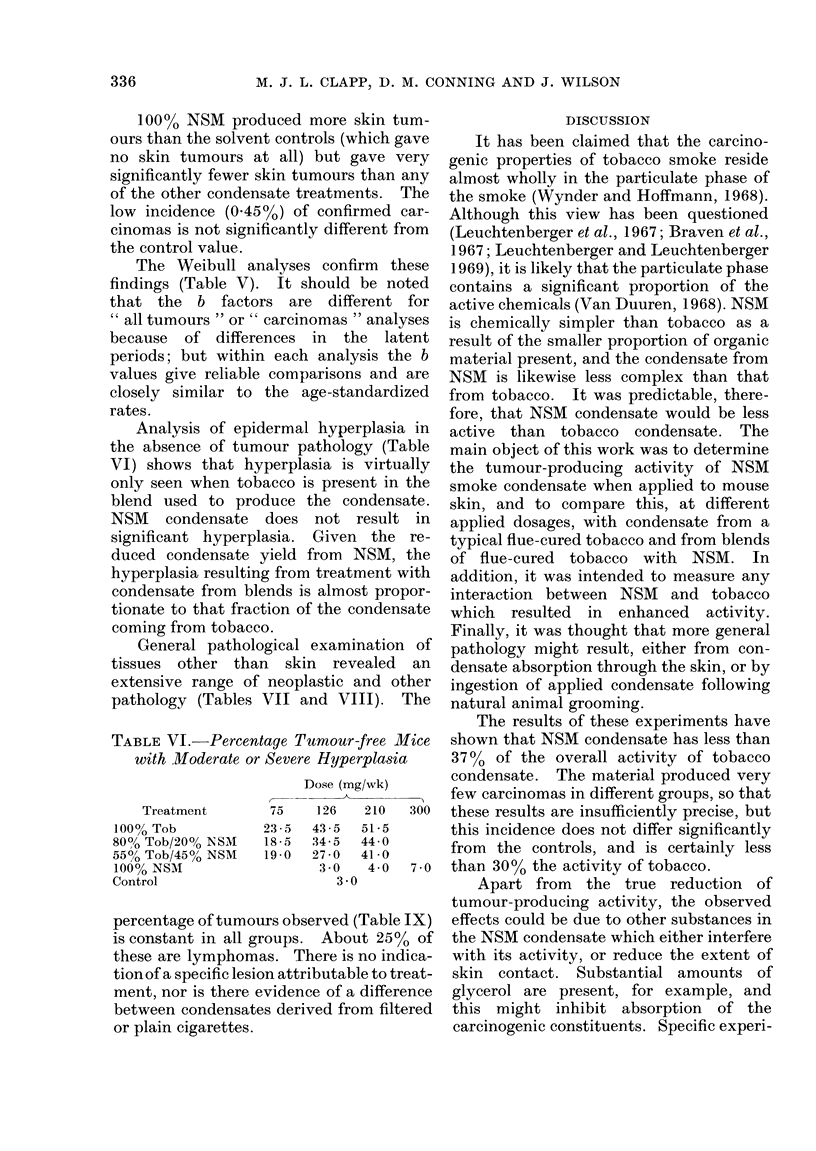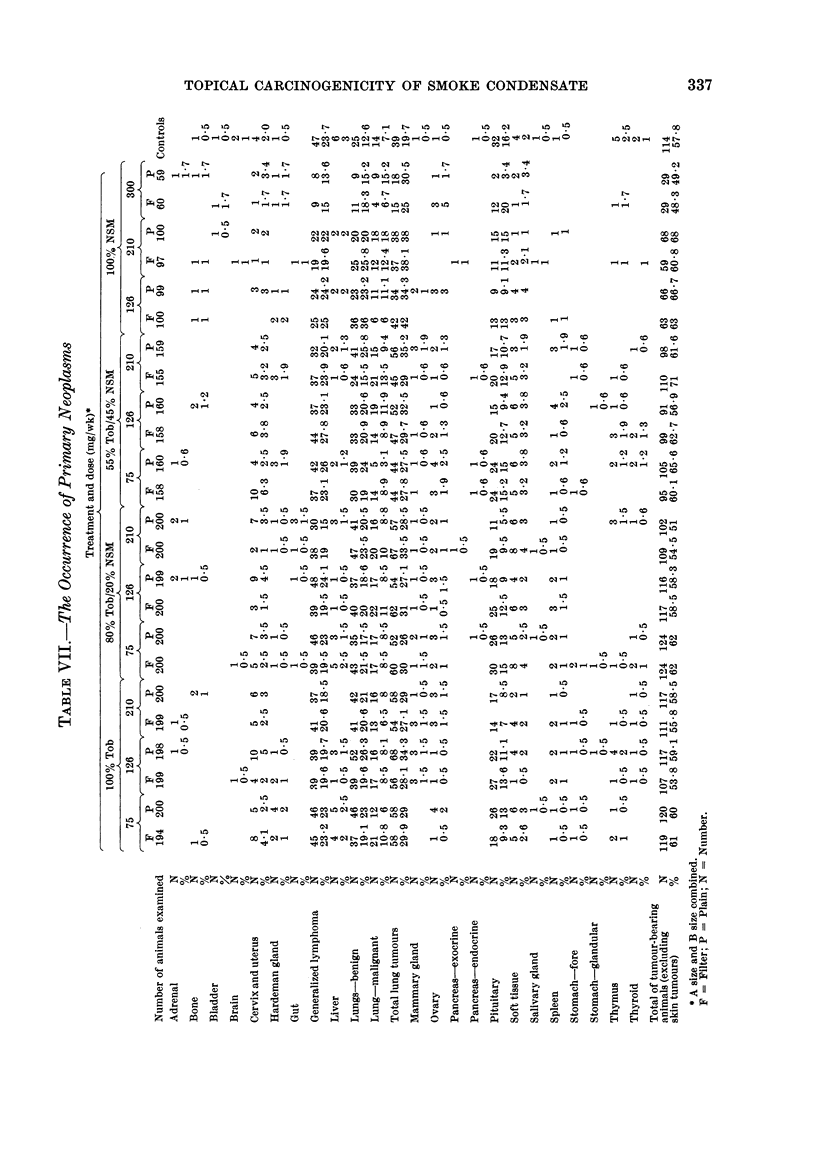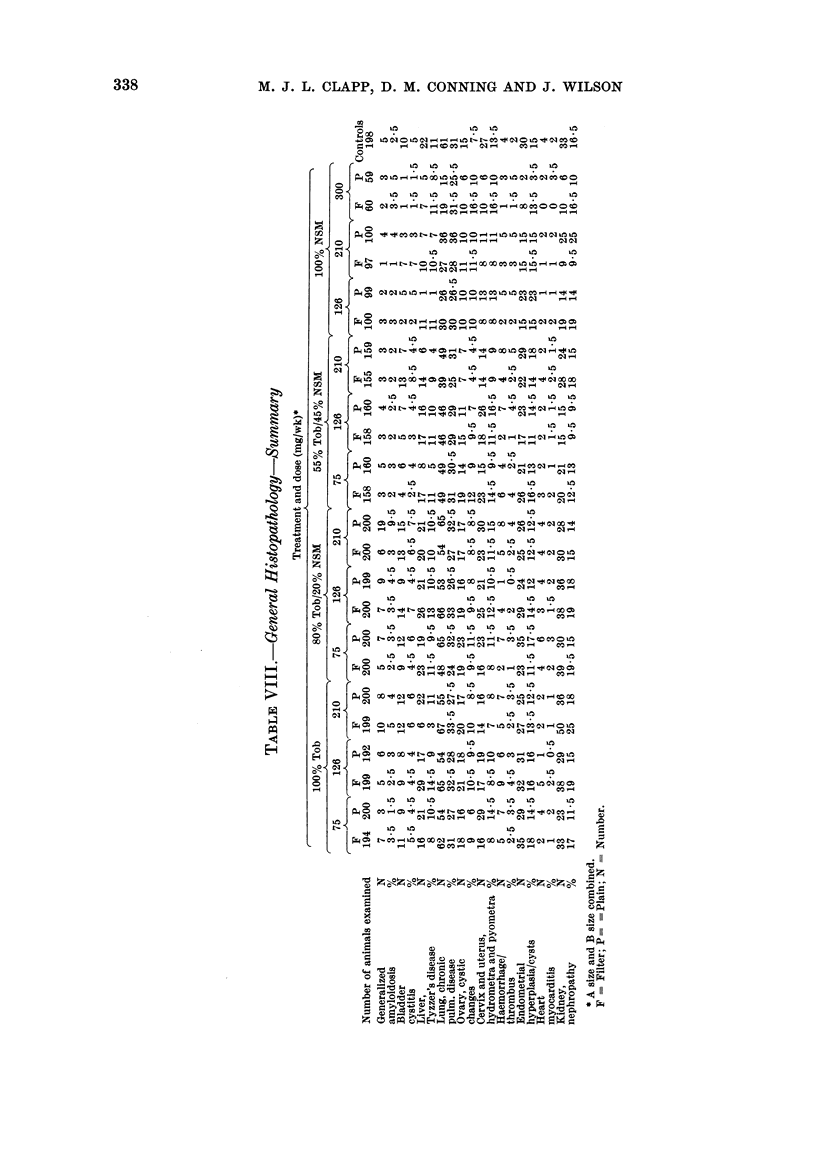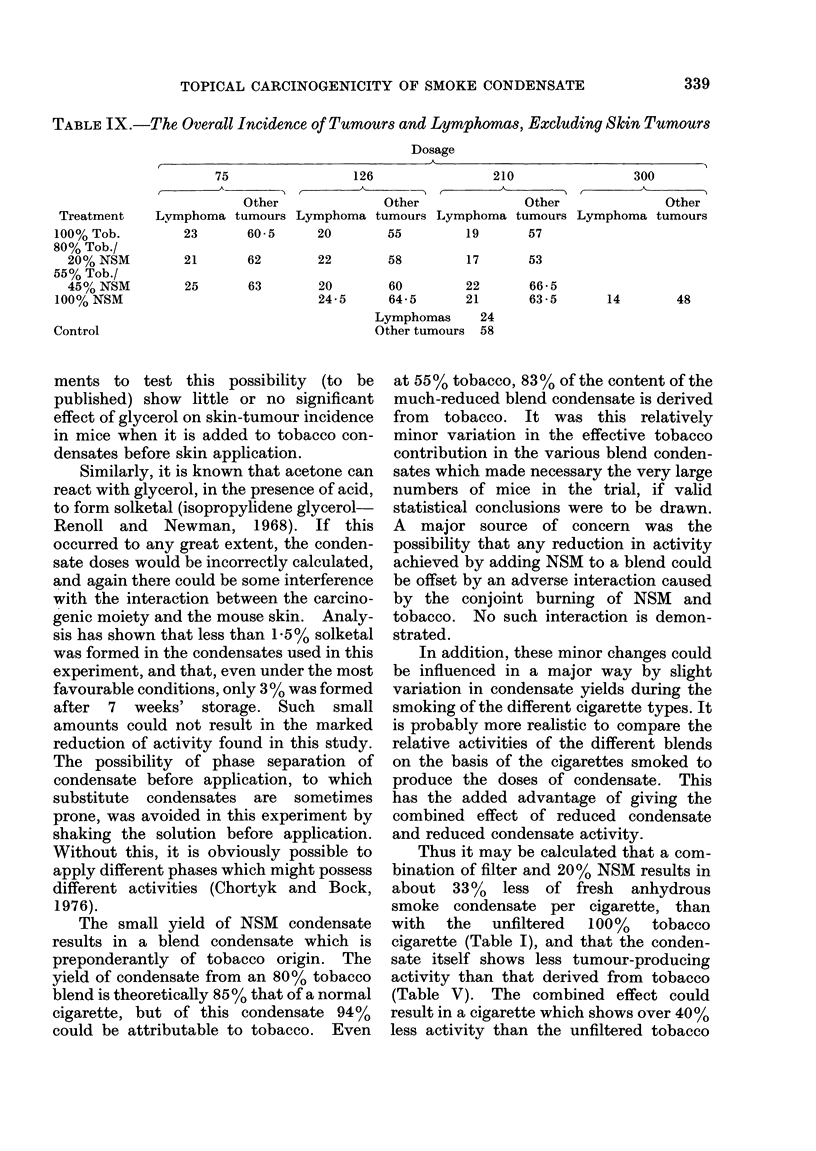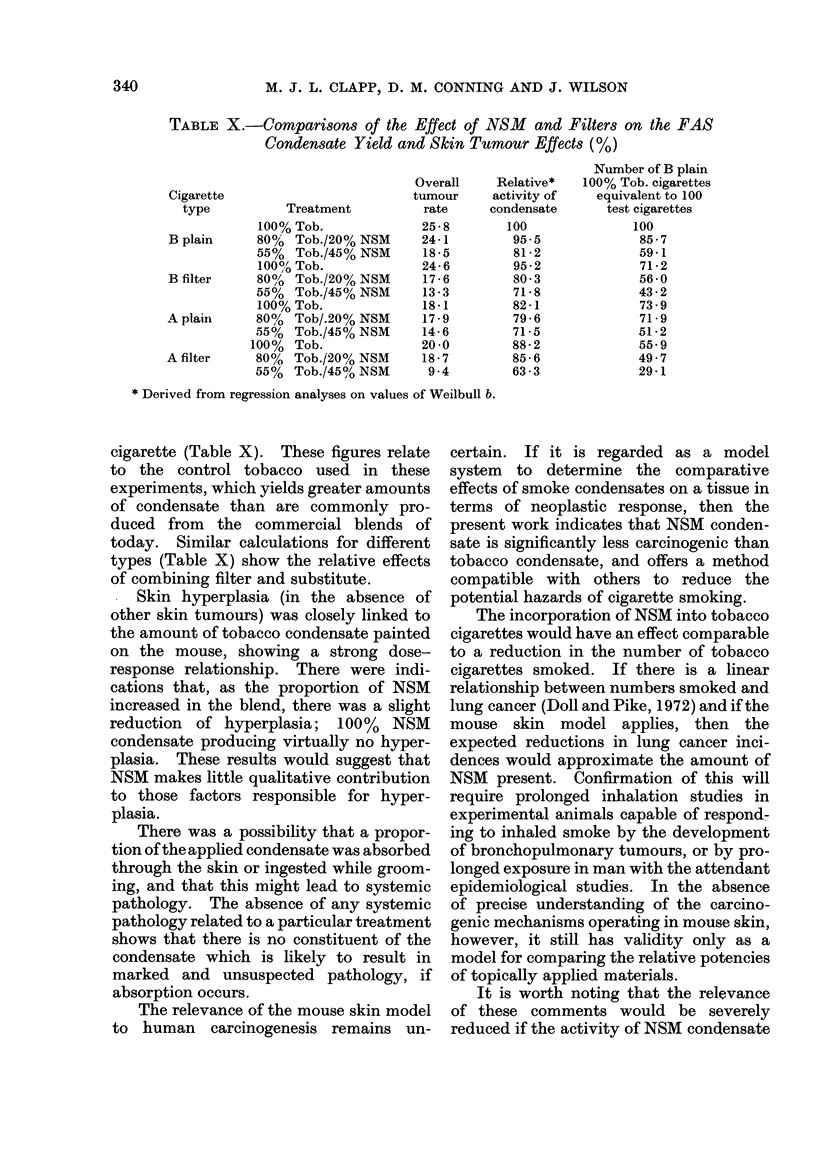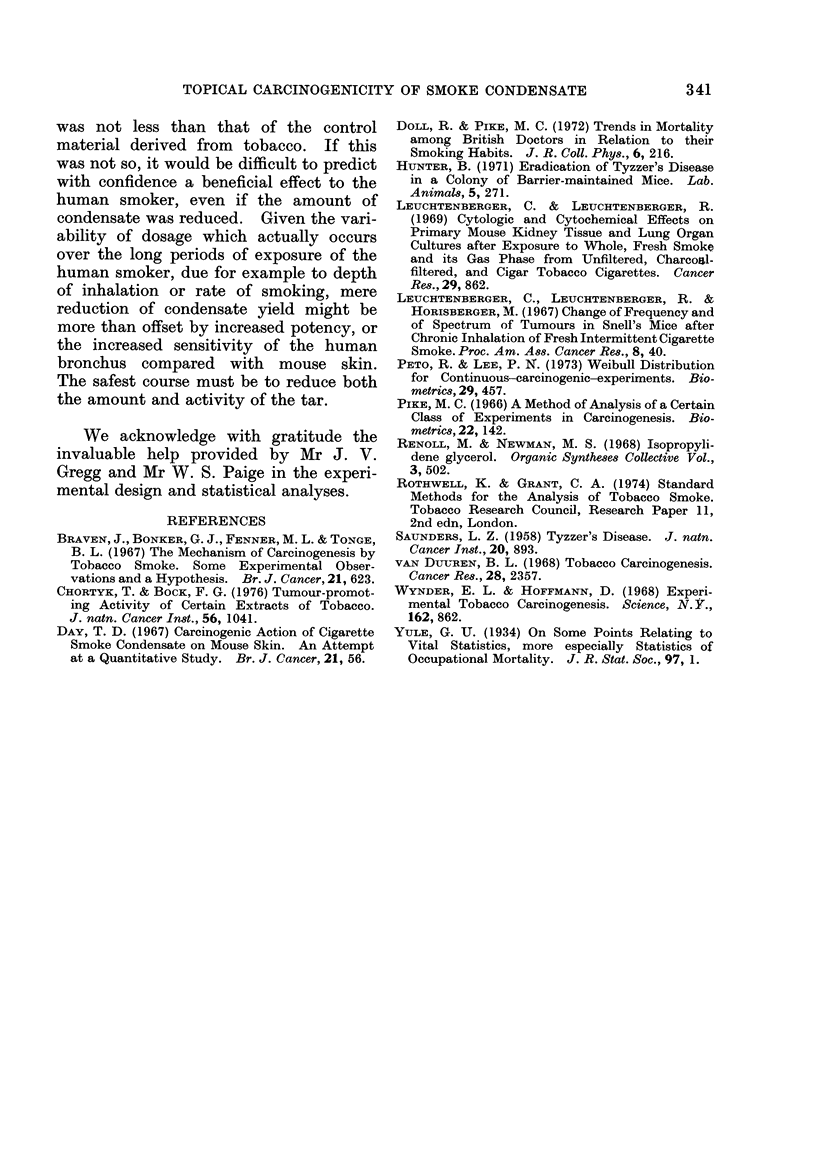# Studies on the local and systemic carcinogenicity of topically applied smoke condensate from a substitute smoking material.

**DOI:** 10.1038/bjc.1977.47

**Published:** 1977-03

**Authors:** M. J. Clapp, D. M. Conning, J. Wilson

## Abstract

The topical carcinogenicity to mouse skin of smoke condensates obtained from a tobacco substitute (NSM), alone or in combination with tobacco, has been compared with condensate from tobacco and with acetone, the solvent used. Sixteen different types of cigarette were used to make the condensates, and the age-standardized results have been analysed according to the Weibull distribution model. The results show that NSM condensate has less than 25% of the potency of tobacco condensate (37% at 95% upper confidence limit), and that condensates from blends of NSM and tobacco are similarly reduced in activity. General pathology analysis failed to reveal abnormalities due to NSM.


*Br. J. Cancer* (1977) **35**, 329.

### STUDIES ON THE LOCAL AND SYSTEMIC CARCINOGENICITY OF TOPICALLY APPLIED SMOKE CONDENSATE FROM A SUBSTITUTE SMOKING MATERIAL

M. J. L. CLAPP, D. M. CONNING AND J. WILSON

*From the Central Toxicology Laboratory, Imperial Chemical Industries Limited, Alderley Park, Macclesfield, Cheshire SK10 4TJ*

Received 23 September 1976 Accepted 12 October 1976

**Summary.—The topical carcinogenicity to mouse skin of smoke condensates obtained from a tobacco substitute (NSM), alone or in combination with tobacco, has been compared with condensate from tobacco and with acetone, the solvent used. Sixteen different types of cigarette were used to make the condensates, and the age-standardized results have been analysed according to the Weibull distribution model. The results show that NSM condensate has less than 25% of the potency of tobacco condensate (37% at 95% upper confidence limit), and that condensates from blends of NSM and tobacco are similarly reduced in activity. General pathology analysis failed to reveal abnormalities due to NSM.**

THE chemical complexity of cigarette smoke precludes the precise attribution of the known carcinogenic effects to a single component or group of components, and animal models are therefore required to assay carcinogenic activity. A model commonly used is the topical application of smoke condensates to mouse skin, and measurement of the incidence of tumours which result. The method has been much used to monitor attempts at reduction of the carcinogenicity of tobacco smoke, and although there is as yet no evidence equating mouse skin response with the human lung response, it is assumed that a direct relationship exists between the ability to induce tumours in mouse skin and in the human bronchial tract.

The experiments described were designed to determine the carcinogenic potential of smoke condensate from a substitute smoking material and from blends of this material with conventional tobacco.

### MATERIAL AND METHODS

*Cigarettes.*—These were 2 sizes, each 70 mm long, but either 23·0 or 25·4 mm in circumference (designated A and B respectively). The cigarettes were either unfiltered or filtered with a 15 mm crimped paper/acetate fibre filter showing 35% nicotine retention. The tobacco used was a commercial blend of flue-cured tobaccos, but contained no crushed stem (Imperial Tobacco Ltd, Bristol).

The substitute was NSM (New Smoking Materials Ltd, Wilmslow) of the following composition:

| | |
|---|---|
| Heat-treated cellulose | 26·9% |
| Sodium carboxy methyl cellulose | 15·0% |
| Glycerol | 6·0% |
| Calcium carbonate | 16·5% |
| Magnesium carbonate | 28·6% |
| Bentonite | 5·0% |
| Ammonium sulphate | 2·0% |

Four different materials were used to prepare condensate:

(1) 100% tobacco

(2) 80% tobacco blended with 20% NSM (80/20 blend)

(3) 55% tobacco blended with 45% NSM (55/45 blend)

(4) 100% NSM.

*Condensate preparation.*—Cigarettes were smoked on a rotary smoking machine (R. W. Mason, Moor Lane, Clevedon, Bristol) similar to that described by Day (1967). The smoking machine operated by connecting in turn each of 24 cigarettes, which were secured in holders situated around a revolving disc, to a vacuum source. The unlighted end of each cigarette was open to the atmosphere between puffs. Cigarettes were lighted by an electrically heated coil, and replaced when individual cigarettes had reached an estimated butt length of 20 mm. The smoking constants used were a conventional puff volume of 35 ml, puff duration of 2 s, and an interval of 1 min (Rothwell and Grant, 1974). Smoke was condensed using a cold impaction trap, and the resultant tar suspended in acetone : water (90 : 10 v/v) mixture. The yields of condensate were expressed in terms of mg of fresh anhydrous smoke (FAS) (Table I). An aliquot of the condensate from each batch of cigarettes was evaporated under reduced pressure on a water bath at 40°C until constant weight was obtained, the residue was returned to the main solution, which was then diluted with the appropriate volume of solvent to give the desired dry weight per unit volume.

The condensates were prepared at weekly intervals and stored at room temperatures until and during use. They were normally between 2 and 3 weeks old when applied to the mice, during which time it is known that the tumour-producing effect of condensate is virtually unchanged (Day, 1967).

The condensate preparations were applied with an " ARH continuous pipetting unit " (Arnold R. Horwell Ltd) through a stainless steel cannula 4 cm long and 2 mm in diameter at the rate of 0·3 $cm^3$ per mouse on 3 days (Monday, Wednesday and Friday) in each week. The animals were not " habituated " to the treatment before starting the experiment. Different doses were achieved by variation in concentration, a fixed volume being applied to each animal.

Control animals were treated with equivalent volumes of solvent only.

*Animals*.—The 4,080 mice used in these experiments were 4–5-week-old-CFLP female mice, a hysterectomy-derived strain of Swiss origin (Carworth-Europe, Alconbury, Huntingdon).

The cages (28·5 × 28 × 10 cm) were constructed at 19-gauge galvanized wire mesh on 3 sides and floor with a solid back, and were suspended over collecting trays. Each rack contained 80 cages, and a total of 16 racks was used for these experiments, 8 racks being housed in each of 2 rooms. The position of each unit was varied within each room to avoid environmental bias from ventilation, light and temperature.

The mice were allocated to cages on arrival, and then distributed amongst the groups on the basis of total body weight per cage, to give equal weight distribution within the groups. They were maintained on pasteurized mouse cubes (Oakes of Congleton, Cheshire) and water *ad libitum*. The animals were kept in a barrier-maintained area throughout the experiment and the animal rooms maintained at a temperature of 72° ± 2°F.

The start was staggered over 13 weeks The experiment was divided to allow equal proportions of each group on the various treatments to commence in the same week. Treatment commenced after 2 weeks' cage acclimatization, when the mice were 6–7 weeks of age. During the second week of

TABLE I.—*Fresh Anhydrous Smoke Condensate (FAS). Yield/cigarette and Cigarettes required for 1 g*

| | | 100% Tobacco | 80% Tobacco/ 20% NSM | 55% Tobacco/ 45% NSM | 100% NSM |
|---|---|---|---|---|---|
| B Plain | FAS mg | 34·1 | 30·6 | 24·8 | 7·1 |
| | Cigs/g | 29 | 33 | 40 | 141 |
| B Filter | FAS mg | 25·5 | 23·8 | 20·5 | 5·5 |
| | Cigs/g | 39 | 42 | 49 | 182 |
| A Plain | FAS mg | 30·7 | 30·8 | 24·4 | 7·6 |
| | Cigs/g | 33 | 32 | 41 | 132 |
| A Filter | FAS mg | 21·6 | 19·8 | 15·7 | 5·5 |
| | Cigs/g | 46 | 51 | 64 | 182 |
| Average | FAS mg | 28 | 26·3 | 21·4 | 6·4 |
| | Cigs/g | 37 | 40 | 49 | 159 |

acclimatization the mice were prepared for the initial treatment by shaving the dorsum from the base of the tail to the nape of the neck, with an Oster A5 electric hair clipper (size 40 blade); this was repeated weekly throughout the study. Care was taken to avoid lacerating the skin, especially if tumours were present. A vacuum attachment over the oscillating teeth of the clipper reduced the dispersion of contaminated hair into the room atmosphere. Very occasionally, the hair required cleaning with acetone to remove inspissated tars prior to clipping.

During the course of the experiment, all data obtained were recorded on cards (Copeland-Chatterson Company Ltd) so that one card represented the history of one mouse.

*Experimental design.*—Preliminary studies showed that NSM condensate has less carcinogenic activity than tobacco condensate, and that relatively few animals are needed to confirm this. A major objective, however,

TABLE II.—*Treatment Groups*

| No. of animals per group | Treatment | FAS dose (mg/wk) | Type of cigarette |
|---|---|---|---|
| 250 | 100% Tobacco | 75 | B plain<br>B filter<br>A plain<br>A filter |
| 250 | 100% Tobacco | 126 | B plain<br>B filter<br>A plain<br>A filter |
| 250 | 100% Tobacco | 210 | B plain<br>B filter<br>A plain<br>A filter |
| 250 | 80% Tob/20% NSM | 75 | B plain<br>B filter<br>A plain<br>A filter |
| 250 | 80% Tob/20% NSM | 126 | B plain<br>B filter<br>A plain<br>A filter |
| 250 | 80% Tob/20% NSM | 210 | B plain<br>B filter<br>A plain<br>A filter |
| 80 | 55% Tob/45% NSM | 75 | B plain<br>B filter<br>A plain<br>A filter |
| 80 | 55% Tob/45% NSM | 126 | B plain<br>B filter<br>A plain<br>A filter |
| 80 | 55% Tob/45% NSM | 210 | B plain<br>B filter<br>A plain<br>A filter |
| 50 | 100% NSM | 126 | B plain<br>B filter<br>A plain<br>A filter |
| 50 | 100% NSM | 210 | B plain<br>B filter<br>A plain<br>A filter |
| 30 | 100% NSM | 300 | B plain<br>B filter<br>A plain<br>A filter |
| 200 | Solvent | — | — |

was to demonstrate with high confidence that no adverse interaction occurs between tobacco and NSM in blends, particularly the 80/20 blend. The numbers of animals vary from group to group, depending on the confidence to be achieved (Table II).

The criteria used were:

(1) To check that blends of tobacco and NSM do not result in a condensate with an increase in skin-tumour-producing activity of 10% or more (1-tail : $\alpha = 5\%$ : $\beta = 10\%$ test) compared with tobacco.
(2) To look for a 25% interaction between tobacco and NSM in skin-tumour-producing activity (1-tail : $\alpha = 5\%$ : $\beta = 5\%$ test).
(3) To check that the skin-tumour-producing activity of NSM condensate is certainly less than 70% that of tobacco, and probably less than 50% (1-tail : $\alpha = 5\%$ : $\beta = 5\%$ test).

The doses of condensate employed were selected to provide as near to a linear dose response as possible. The low skin-tumour-producing potential of NSM condensate necessitated larger dosage with this material.

### *Observations*

*Body weight.*—Individual mice were weighed initially, and then weekly during the first 12 weeks, and thereafter at fortnightly intervals until death.

*Food consumption.*—Food consumption per cage of 10 mice was recorded weekly for the first 12 weeks.

*Clinical observations.*—All animals were checked daily, and any abnormality in behaviour or physical condition recorded. Once a week, special attention was given to the skin, and a count and classification made of any tumours present; the exact site and date of appearance were noted on the mouse record card. Papillomas, including suspected sebaceous adenomas, were recorded when they appeared to be greater than 1 mm$^3$ in size, and they had been present for at least 2 consecutive weeks. Papillomas were classified as suspected carcinomas when there was a swelling of the dermis beneath the papilloma or the papilloma became " fixed " suggesting a (tumorous) connection between the skin and underlying muscle; or when the papilloma had sloughed at the centre to give rise to an ulcerated crater with rolled edges. Regression was recorded when a papilloma which had been present for more than 2 weeks completely disappeared. These observations were made on all animals in the experiment.

*Pathology.*—A full *post-mortem* examination was made on all animals. Any mouse which became moribund or distressed due to disease (including tumour) during the course of the experiment was killed and examined. Those mice which died spontaneously were examined as soon as possible, and always within 24 h of death. Where severe autolysis had occurred, only skin and macroscopically abnormal tissues were fixed for histopathological examination. Rarely, all tissues were lost because of complete cannibalism (0·5% of all deaths).

The skin histopathological examination is reported for the control animals and all treated with condensates. Histopathology of all other tissues taken was done on all the animals in the control group, on 100 animals chosen by random selection from groups which contained 250 animals, and on all animals from other groups, provided the tissues were not autolysed or cannibalized. The total number of animals eventually studied was 4,052 out of the possible 4,080.

During the course of a *post-mortem* examination, sections of the following tissues were preserved in formol-corrosive for histopathological examination: adrenal, caecum, colon, duodenum, heart, ileum, jejunum, kidney, liver, lungs, (having first been inflated with formol saline), ovary, pancreas, pituitary, salivary gland, spleen, stomach, thymus (where it had not involuted), thyroid, urinary bladder, uterus, voluntary muscle and any abnormal tissue. The brain was included in the case of suspected tumours, or where abnormal behaviour had been observed before death. Sections were cut at 5 $\mu$m, stained with haematoxylin and eosin, and special stains where appropriate. Lymph nodes (abdominal, axillary, cervical, inguinal, mesenteric, renal and thymic) were examined during the course of the *post-mortem* examination, but not preserved unless apparently abnormal.

Sections of skin including some normal tissue, and all tumours inside or outside the treated area, were preserved in Bouin's fixative for histopathological examination.

To minimize operator bias in these and *post-mortem* procedures, the training of all experimental assistants was managed by one person, and each assistant at different times was involved in all the procedures, and with each group of animals.

Histopathology of skin on all animals in the experiment was classified as follows:

0 Normal skin.
1 Tumours which would not normally be classified as skin tumours, *e.g.* mast cell tumours and secondary lymphoma.
2 Hyperkeratosis and hyperplasia.
3 Papilloma and other benign tumours of epithelial origin, including sebaceous adenoma.
4 Carcinoma I (*in situ*) or tumour in which there is no evidence of infiltration but some cells show changes characteristic of malignancy.
5 Carcinoma II: infiltrating dermis but not into muscle.
6 Carcinoma III: a tumour infiltrating muscle.
7 Benign tumours of connective tissue.
8 Sarcoma: malignant tumours of connective tissue.

An analysis of the incidence of hyperplasia was based on the following criteria:

Normal: up to 3 layers of nucleated cells in the epithelium.
Moderate hyperplasia: 3–4 layers of nucleated cells.
Severe hyperplasia: $>4$ layers of nucleated cells.

The slides were examined by a group of 6 pathologists, and the diagnoses coded for computer storage. The pathologists agreed the terminology to be used, but there was no attempt to standardize the individual diagnoses. The slides were allocated to individual pathologists by a random selection procedure which ensured that each of them received material from mice in all experimental groups.

Skin-tumour incidence was subjected to statistical analysis both for all tumour-bearing animals, and for animals with proven carcinomas. Analyses were of 2 types: the classical age-standardization technique, which gives a percentage tumour incidence corrected for varying mortality patterns (Yule, 1934; Pike, 1966; Peto and Lee, 1973) and a mathematical modelling technique based upon the Weibull distribution (Pike, 1966; Peto and Lee, 1973). This technique gives a parameter $b$ which measures the intensity of the tumour-producing response to treatment; $b$ contains a scaling factor which is different for the " all tumour " and " confined carcinomas " analysis, but within either analysis the $b$ values give reliable comparisons of treatment effects. The scaling factor is due to measurement of " age to tumour " in weeks. An average of " age to tumour " is raised to the power of 3·5 for all tumours and 10 for carcinomas, giving scaling factors of $10^7$ and $10^{23}$ respectively.

The $b$ factors calculated were almost proportional to the corresponding standardized rates, and interpretation of activity is virtually the same for both criteria.

### RESULTS

### *Clinical observations*

Evidence of nicotine poisoning (tremors, and clonic contractions on external stimulus, followed by a period of subdued behaviour) was observed in the majority of mice receiving the high doses from 100% tobacco or 80% tobacco/20% NSM mixture during the first few weeks of the experiment. This resulted in the death of 8 mice receiving the high-dose condensate from size A plain tobacco cigarettes. The symptoms of nicotine poisoning occurred within 10 min of dosing, and lasted for several hours. By Week 8 the evidence of nicotine poisoning was minimal; animals had become acclimatized to their respective treatments.

Also during the first 8 weeks, some matting of the fur occurred, especially on 100% tobacco and 80% tobacco/20% NSM mixture; this caused irritation, and increased scratching of the painted area occurred.

An outbreak of Tyzzer's disease occurred between 34 and 49 weeks (depending on the staggered start). A total of 488 mice was autopsied over a period of 6 weeks and, of these, 128 showed typical Tyzzer's lesions of the liver. To prevent the outbreak reaching epidemic proportions and the potential loss of the experiment, all mice were treated with oxytetracycline (25 mg/kg, " Imperacin ") in the drinking water for 5 days. Oxytetracycline has previously been found to be effective in

TABLE III.—*Percentage of Mice Bearing Skin Tumours*

| Group | | Observed FAS dose (mg/wk) 75 | 126 | 210 | 300 | Mean | Age-standardized (Yule) FAS dose (mg/wk) 75 | 126 | 210 | 300 | Mean |
|---|---|---|---|---|---|---|---|---|---|---|---|
| 100% Tob | B plain | 10·8 | 27·2 | 44·0 | | 23·54 | 6·6 | 21·8 | 49·0 | | 22·13 |
| | B filter | 10·0 | 20·9 | 44·4 | | | 6·6 | 15·7 | 51·7 | | |
| | A plain | 7·6 | 18·4 | 36·7 | | | 4·8 | 13·3 | 36·1 | | |
| | A filter | 8·8 | 14·9 | 38·8 | | | 7·1 | 11·3 | 41·5 | | |
| | Mean | 9·3 | 20·35 | 41·0 | | | 6·3 | 15·5 | 44·6 | | |
| Av. cigs smoked* | | 2·8 | 4·7 | 7·8 | | | 2·8 | 4·6 | 7·7 | | |
| 80% Tob/ 20% NSM | B plain | 10·4 | 24·8 | 44·8 | | 21·35 | 6·6 | 18·2 | 47·6 | | 19·58 |
| | B filter | 10·0 | 15·3 | 33·7 | | | 6·9 | 12·5 | 33·3 | | |
| | A plain | 8·4 | 11·6 | 36·4 | | | 6·6 | 9·3 | 37·8 | | |
| | A filter | 7·2 | 16·8 | 36·8 | | | 5·4 | 13·4 | 37·4 | | |
| | Mean | 9·0 | 17·1 | 37·9 | | | 6·4 | 13·4 | 39·0 | | |
| Av. cigs smoked* | | 3·0 | 5·0 | 8·0 | | | 3·0 | 5·0 | 8·0 | | |
| 55% Tob/ 45% NSM | B plain | 6·3 | 16·3 | 40·0 | | 17·34 | 4·8 | 11·3 | 39·4 | | 13·95 |
| | B filter | 10·1 | 13·8 | 32·5 | | | 5·0 | 10·2 | 24·8 | | |
| | A plain | 10·0 | 19·0 | 27·5 | | | 6·7 | 16·7 | 20·4 | | |
| | A filter | 1·3 | 8·8 | 22·5 | | | 0·8 | 7·1 | 20·2 | | |
| | Mean | 6·9 | 14·5 | 30·6 | | | 4·3 | 11·3 | 26·2 | | |
| Av. cigs smoked* | | 3·7 | 6·2 | 10·3 | | | 3·7 | 6·2 | 10·3 | | |
| 100% NSM | B plain | | 2·0 | 6·0 | 13·3 | 6·63 | | 1·6 | 4·5 | 13·0 | 5·13 |
| | B filter | | 4·0 | 6·0 | 13·3 | | | 2·3 | 3·1 | 9·1 | |
| | A plain | | 4·0 | 8·0 | 10·3 | | | 2·7 | 6·2 | 11·2 | |
| | A filter | | 2·0 | 4·0 | 6·7 | | | 0·9 | 3·0 | 3·9 | |
| | Mean | | 3·0 | 6·0 | 10·9 | | | 1·9 | 4·2 | 9·3 | |
| Av. cigs smoked* | | | 20·0 | 33·4 | 47·7 | | | 20·0 | 33·4 | 47·7 | |

All control values are zero.
* Average cigarettes smoked: number of cigarettes to produce the indicated mean FAS condensate dose.

the control of Tyzzer's disease (Hunter, 1971). It has been suggested that Tyzzer's disease is endemic in certain strains of mice (Saunders, 1958), but an outbreak only occurs when the mice are subjected to a stress factor. No correlation could be found between this episode and the subsequent tumour response. A second minor outbreak occurred between weeks 60 and 75, and again the infection was controlled by treatment with oxytetracycline.

The overall skin-tumour response (Table III) shows that there is a reduction of tumour response when the proportion of NSM present is increased, and that there is a well-defined dose–response relationship, the tumour yield increasing with dosage of condensate. There is no departure from these relationships when any of the variables are considered on the basis of log dose–log response effect, and it is therefore legitimate to treat the groups as replicates when studying the 4 cigarette types.

NSM condensate produced about 25% of the total number of skin tumours produced by a similar dose of tobacco condensate. It caused less than 5% of the equivalent total of malignant tumours (Table IV). Significant reductions of activity per unit dose of condensate were also observed with the blends (80/20, $P < 0{\cdot}05$; 55/45, $P < 0{\cdot}001$). In terms of dose required to produce the same tumour response, NSM showed 31% of the total tumour generation of tobacco (*i.e.*, 31 parts of tobacco is equivalent to 100 parts NSM), and about 22% of the malignant tumour activity (37% and 30% respectively at the upper confidence limits of 95%). The 80/20 blend showed 94% and 97% activity respectively on this basis, and the 55/45 blend 79% and 75%.

TABLE IV.—*Percentage Mice Bearing Confirmed Carcinomas*

| Group | | Observed FAS dose (mg/wk) 75 | 126 | 210 | 300 | Mean | Age standardized (Yule) FAS dose (mg/wk) 75 | 126 | 210 | 300 | Mean |
|---|---|---|---|---|---|---|---|---|---|---|---|
| 100% Tob | B plain | 3·2 | 8·4 | 24·8 | | | 1·3 | 4·6 | 20·3 | | |
| | B filter | 1·2 | 7·6 | 21·2 | | 9·86 | 0·7 | 3·9 | 15·7 | | 6·77 |
| | A plain | 1·6 | 6·8 | 18·7 | | | 0·8 | 3·5 | 11·7 | | |
| | A filter | 1·2 | 5·2 | 18·4 | | | 0·8 | 2·9 | 14·4 | | |
| | Mean | 1·8 | 7·0 | 20·8 | | | 0·9 | 3·7 | 15·7 | | |
| 80% Tob/ 20% NSM | B plain | 2·8 | 7·6 | 26·0 | | | 1·8 | 3·5 | 19·8 | | |
| | B filter | 1·6 | 2·0 | 16·1 | | 8·81 | 0·9 | 1·5 | 12·1 | | 6·43 |
| | A plain | 2·4 | 5·6 | 19·2 | | | 1·6 | 3·7 | 15·8 | | |
| | A filter | 0·8 | 6·4 | 15·2 | | | 0·6 | 3·8 | 12·0 | | |
| | Mean | 1·9 | 5·4 | 19·1 | | | 1·2 | 3·1 | 14·9 | | |
| 55% Tob/ 45% NSM | B plain | 2·5 | 2·5 | 13·8 | | | 1·2 | 1·9 | 7·8 | | |
| | B filter | 0 | 3·8 | 12·5 | | 5·12 | 0 | 2·8 | 6·5 | | 3·08 |
| | A plain | 1·3 | 3·8 | 10·0 | | | 0·8 | 2·9 | 5·4 | | |
| | A filter | 0 | 2·5 | 8·8 | | | 0 | 1·5 | 6·2 | | |
| | Mean | 0·95 | 3·2 | 11·3 | | | 0·5 | 2·3 | 6·5 | | |
| 100% NSM | B plain | | 0 | 0 | 0 | | | 0 | 0 | 0 | |
| | B filter | | 0 | 0 | 0 | 0·45 | | 0 | 0 | 0 | 0·31 |
| | A plain | | 0 | 0 | 3·4 | | | 0 | 0 | 2·6 | |
| | A filter | | 0 | 2·0 | 0 | | | 0 | 1·1 | 0 | |
| | Mean | | 0 | 0·5 | 0·9 | | | 0 | 0·3 | 0·7 | |

All control values are zero.

TABLE V.—*Weibull* b *Constants*‡

| Group | | All tumours* FAS dose (mg/wk) 75 | 126 | 210 | 300 | Mean | Confirmed carcinomas† FAS dose (mg/wk) 75 | 126 | 210 | 300 | Mean |
|---|---|---|---|---|---|---|---|---|---|---|---|
| 100% Tob | B plain | 1·82 | 5·49 | 12·35 | | | 2·45 | 7·21 | 31·62 | | |
| | B filter | 1·65 | 3·84 | 13·05 | | 5·59 | 1·00 | 6·19 | 25·43 | | 10·60 |
| | A plain | 1·25 | 3·42 | 9·07 | | | 1·20 | 5·86 | 18·17 | | |
| | A filter | 1·75 | 2·77 | 10·72 | | | 1·24 | 4·51 | 22·37 | | |
| | Mean | 1·62 | 3·88 | 11·30 | | | 1·47 | 5·94 | 24·40 | | |
| 80% Tob/ 20% NSM | B plain | 1·72 | 4·53 | 12·11 | | | 2·14 | 6·0 | 29·28 | | |
| | B filter | 1·67 | 3·09 | 8·46 | | 4·91 | 1·21 | 2·18 | 19·04 | | 9·83 |
| | A plain | 1·66 | 2·32 | 9·09 | | | 2·55 | 8·96 | 24·28 | | |
| | A filter | 1·39 | 3·34 | 9·40 | | | 0·80 | 5·60 | 18·80 | | |
| | Mean | 1·61 | 3·32 | 9·81 | | | 1·68 | 4·93 | 22·88 | | |
| 80% Tob/ 20% NSM | B plain | 1·16 | 2·86 | 9·61 | | | 2·37 | 1·91 | 13·40 | | |
| | B filter | 1·44 | 2·62 | 6·45 | | 3·50 | 0 | 4·22 | 9·92 | | 4·91 |
| | A plain | 1·41 | 4·25 | 5·14 | | | 0·95 | 4·58 | 8·80 | | |
| | A filter | 0·23 | 1·75 | 5·03 | | | 0 | 2·46 | 10·30 | | |
| | Mean | 1·06 | 2·87 | 6·56 | | | 0·83 | 3·29 | 10·61 | | |
| 100% NSM | B plain | | 0·31 | 1·14 | 3·45 | | | 0 | 0 | 0 | |
| | B filter | | 0·71 | 0·90 | 2·66 | 1·40 | | 0 | 0 | 0 | 0·61 |
| | A plain | | 0·72 | 1·57 | 2·32 | | | 0 | 0 | 4·94 | |
| | A filter | | 0·38 | 0·85 | 1·28 | | | 0 | 2·42 | 0 | |
| | Mean | | 0·53 | 1·11 | 2·43 | | | 0 | 0·60 | 1·24 | |

No malignant tumours occurred in control animals.
* Values given are $b \times 10^{7}$.
† Values given are $b \times 10^{23}$.
‡ Weibull distribution model of the incidence of tumours over the life-time of mice is proportion of tumour-bearing animals = exp $(b(t\text{-}w)^k)$, where $t$ = time at which the proportion of tumour-bearing mice is calculated.

Analysis indicated the common use of the following for each of the above 16 groups

| | All tumours | Confirmed carcinomas |
|---|---|---|
| Weibull constant $w$ | 8·935 | −34·615 |
| Weibull constant $k$ | 3·094 | 10·0 |

100% NSM produced more skin tumours than the solvent controls (which gave no skin tumours at all) but gave very significantly fewer skin tumours than any of the other condensate treatments. The low incidence (0·45%) of confirmed carcinomas is not significantly different from the control value.

The Weibull analyses confirm these findings (Table V). It should be noted that the *b* factors are different for " all tumours " or " carcinomas " analyses because of differences in the latent periods; but within each analysis the *b* values give reliable comparisons and are closely similar to the age-standardized rates.

Analysis of epidermal hyperplasia in the absence of tumour pathology (Table VI) shows that hyperplasia is virtually only seen when tobacco is present in the blend used to produce the condensate. NSM condensate does not result in significant hyperplasia. Given the reduced condensate yield from NSM, the hyperplasia resulting from treatment with condensate from blends is almost proportionate to that fraction of the condensate coming from tobacco.

General pathological examination of tissues other than skin revealed an extensive range of neoplastic and other pathology (Tables VII and VIII). The percentage of tumours observed (Table IX) is constant in all groups. About 25% of these are lymphomas. There is no indication of a specific lesion attributable to treatment, nor is there evidence of a difference between condensates derived from filtered or plain cigarettes.

TABLE VI.—*Percentage Tumour-free Mice with Moderate or Severe Hyperplasia*

| | Dose (mg/wk) | | | |
|---|---|---|---|---|
| Treatment | 75 | 126 | 210 | 300 |
| 100% Tob | 23·5 | 43·5 | 51·5 | |
| 80% Tob/20% NSM | 18·5 | 34·5 | 44·0 | |
| 55% Tob/45% NSM | 19·0 | 27·0 | 41·0 | |
| 100% NSM | | 3·0 | 4·0 | 7·0 |
| Control | | 3·0 | | |

DISCUSSION

It has been claimed that the carcinogenic properties of tobacco smoke reside almost wholly in the particulate phase of the smoke (Wynder and Hoffmann, 1968). Although this view has been questioned (Leuchtenberger *et al.*, 1967; Braven *et al.*, 1967; Leuchtenberger and Leuchtenberger 1969), it is likely that the particulate phase contains a significant proportion of the active chemicals (Van Duuren, 1968). NSM is chemically simpler than tobacco as a result of the smaller proportion of organic material present, and the condensate from NSM is likewise less complex than that from tobacco. It was predictable, therefore, that NSM condensate would be less active than tobacco condensate. The main object of this work was to determine the tumour-producing activity of NSM smoke condensate when applied to mouse skin, and to compare this, at different applied dosages, with condensate from a typical flue-cured tobacco and from blends of flue-cured tobacco with NSM. In addition, it was intended to measure any interaction between NSM and tobacco which resulted in enhanced activity. Finally, it was thought that more general pathology might result, either from condensate absorption through the skin, or by ingestion of applied condensate following natural animal grooming.

The results of these experiments have shown that NSM condensate has less than 37% of the overall activity of tobacco condensate. The material produced very few carcinomas in different groups, so that these results are insufficiently precise, but this incidence does not differ significantly from the controls, and is certainly less than 30% the activity of tobacco.

Apart from the true reduction of tumour-producing activity, the observed effects could be due to other substances in the NSM condensate which either interfere with its activity, or reduce the extent of skin contact. Substantial amounts of glycerol are present, for example, and this might inhibit absorption of the carcinogenic constituents. Specific experi-

### TABLE VII.—*The Occurrence of Primary Neoplasms*

Treatment and dose (mg/wk)*

| | | 100% Tob | | | | | | 80% Tob/20% NSM | | | | | | 55% Tob/45% NSM | | | | | | 100% NSM | | | | | | |
|---|---|---|---|---|---|---|---|---|---|---|---|---|---|---|---|---|---|---|---|---|---|---|---|---|---|---|
| | | 75 | | 126 | | 210 | | 75 | | 126 | | 210 | | 75 | | 126 | | 210 | | 126 | | 210 | | 300 | | |
| | | F | P | F | P | F | P | F | P | F | P | F | P | F | P | F | P | F | P | F | P | F | P | F | P | |
| Number of animals examined | | 194 | 200 | 199 | 198 | 199 | 200 | 200 | 200 | 200 | 199 | 200 | 200 | 158 | 160 | 158 | 160 | 155 | 159 | 100 | 99 | 97 | 100 | 60 | 59 | Controls |
| Adrenal | N | | | | 1 | 1 | | | | | 2 | | 2 | | 1 | | | | | | | | | | 1 | |
| | % | | | | 0·5 | 0·5 | | | | | 1 | | 1 | | 0·6 | | | | | | | | | | 1·7 | |
| Bone | N | 1 | | | | | 2 | | | | 1 | | | | | | 2 | | | 1 | 1 | 1 | | | 1 | 1 |
| | % | 0·5 | | | | | 1 | | | | 0·5 | | | | | | 1·2 | | | 1 | 1 | 1 | | | 1·7 | 0·5 |
| Bladder | N | | | | | | | | | | | | | | | | | | | | | | 1 | 1 | | 1 |
| | % | | | | | | | | | | | | | | | | | | | | | | 0·5 | 1·7 | | 0·5 |
| Brain | N | | | 1 | | | | 1 | | | | | | | | | | | | | | 1 | | | | 2 |
| | % | | | 0·5 | | | | 0·5 | | | | | | | | | | | | | | 1 | | | | 1 |
| Cervix and uterus | N | 8 | 5 | 4 | 10 | 5 | 6 | 5 | 7 | 3 | 9 | 2 | 7 | 10 | 4 | 6 | 4 | 5 | 4 | | 3 | 1 | 2 | 1 | 2 | 4 |
| | % | 4·1 | 2·5 | 2 | 5 | 2·5 | 3 | 2·5 | 3·5 | 1·5 | 4·5 | 1 | 3·5 | 6·3 | 2·5 | 3·8 | 2·5 | 3·2 | 2·5 | | 3 | 1 | 2 | 1·7 | 3·4 | 2·0 |
| Hardeman gland | N | 2 | 4 | 2 | 1 | | | 1 | 1 | | | 1 | 1 | | 3 | | | 3 | | 2 | 1 | | | 1 | 1 | 1 |
| | % | 1 | 2 | 1 | 0·5 | | | 0·5 | 0·5 | | | 0·5 | 0·5 | | 1·9 | | | 1·9 | | 2 | 1 | | | 1·7 | 1·7 | 0·5 |
| Gut | N | | | | | | | 1 | | | 1 | 1 | 3 | | | | | | | | | 1 | | | | |
| | % | | | | | | | 0·5 | | | 0·5 | 0·5 | 1·5 | | | | | | | | | 1 | | | | |
| Generalized lymphoma | N | 45 | 46 | 39 | 39 | 41 | 37 | 39 | 46 | 39 | 48 | 38 | 30 | 37 | 42 | 44 | 37 | 37 | 32 | 25 | 24 | 19 | 22 | 9 | 8 | 47 |
| | % | 23·2 | 23 | 19·6 | 19·7 | 20·6 | 18·5 | 19·5 | 23 | 19·5 | 24·1 | 19 | 15 | 23·1 | 26 | 27·8 | 23·1 | 23·9 | 20·1 | 25 | 24·2 | 19·6 | 22 | 15 | 13·6 | 23·7 |
| Liver | N | 4 | 5 | 1 | 3 | | | 5 | 3 | 1 | 1 | | 3 | | 2 | | | 1 | 2 | | 2 | | 2 | | | 6 |
| | % | 2 | 2·5 | 0·5 | 1·5 | | | 2·5 | 1·5 | 0·5 | 0·5 | | 1·5 | | 1·2 | | | 0·6 | 1·3 | | 2 | | 2 | | | 3 |
| Lungs—benign | N | 37 | 46 | 39 | 52 | 41 | 42 | 43 | 35 | 40 | 37 | 47 | 41 | 30 | 39 | 33 | 33 | 24 | 41 | 36 | 23 | 25 | 20 | 11 | 9 | 25 |
| | % | 19·1 | 23 | 19·6 | 26·3 | 20·6 | 21 | 21·5 | 17·5 | 20 | 18·6 | 23·5 | 20·5 | 19 | 24 | 20·9 | 20·6 | 15·5 | 25·8 | 36 | 23·2 | 25·8 | 20 | 18·3 | 15·2 | 12·6 |
| Lung—malignant | N | 21 | 12 | 17 | 16 | 13 | 16 | 17 | 17 | 22 | 17 | 20 | 16 | 14 | 5 | 14 | 19 | 21 | 15 | 6 | 11 | 12 | 18 | 4 | 9 | 14 |
| | % | 10·8 | 6 | 8·5 | 8·1 | 6·5 | 8 | 8·5 | 8·5 | 11 | 8·5 | 10 | 8·8 | 8·9 | 3·1 | 8·9 | 11·9 | 13·5 | 9·4 | 6 | 11·1 | 12·4 | 18 | 6·7 | 15·2 | 7·1 |
| Total lung tumours | N | 58 | 58 | 56 | 68 | 54 | 58 | 60 | 52 | 62 | 54 | 67 | 57 | 44 | 44 | 47 | 52 | 45 | 56 | 42 | 34 | 37 | 38 | 15 | 18 | 39 |
| | % | 29·9 | 29 | 28·1 | 34·3 | 27·1 | 29 | 30 | 26 | 31 | 27·1 | 33·5 | 28·5 | 27·8 | 27·5 | 29·7 | 32·5 | 29 | 35·2 | 42 | 34·3 | 38·1 | 38 | 25 | 30·5 | 19·7 |
| Mammary gland | N | | | 3 | 3 | 3 | 1 | 1 | 2 | 1 | 1 | 1 | 1 | 1 | 1 | 1 | | 1 | 3 | | 2 | | | | | 1 |
| | % | | | 1·5 | 1·5 | 1·5 | 0·5 | 1·5 | 1 | 0·5 | 0·5 | 0·5 | 0·5 | | 0·6 | 0·6 | | 0·6 | 1·9 | | 1 | | | | | 0·5 |
| Ovary | N | 1 | 4 | 1 | 1 | 3 | 3 | 2 | 3 | 1 | 3 | 2 | 2 | 3 | 4 | 2 | 1 | 1 | 2 | | 3 | | 1 | 3 | 1 | 1 |
| | % | 0·5 | 2 | 0·5 | 0·5 | 1·5 | 1·5 | 1 | 1·5 | 0·5 | 1·5 | 1 | 1 | 1·9 | 2·5 | 1·3 | 0·6 | 0·6 | 1·3 | | 3 | | 1 | 5 | 1·7 | 0·5 |
| Pancreas—exocrine | N | | | | | | | | | | | 1 | | | | | | | | | | 1 | | | | |
| | % | | | | | | | | | | | 0·5 | | | | | | | | | | 1 | | | | |
| Pancreas—endocrine | N | | | | | | | | 1 | | 1 | | | 1 | 1 | | | 1 | | | | | | | | 1 |
| | % | | | | | | | | 0·5 | | 0·5 | | | 0·6 | 0·6 | | | 0·6 | | | | | | | | 0·5 |
| Pituitary | N | 18 | 26 | 27 | 22 | 14 | 17 | 30 | 26 | 25 | 18 | 19 | 11 | 24 | 24 | 20 | 15 | 20 | 17 | 13 | 9 | 11 | 15 | 12 | 2 | 32 |
| | % | 9·3 | 13 | 13·6 | 11·1 | 7 | 8·5 | 15 | 13 | 12·5 | 9 | 9·5 | 5·5 | 15·2 | 15 | 12·7 | 9·4 | 12·9 | 10·7 | 13 | 9·1 | 11·3 | 15 | 20 | 3·4 | 16·2 |
| Soft tissue | N | 5 | 6 | 1 | 4 | 4 | 2 | 8 | 5 | 6 | 4 | 8 | 6 | 5 | 6 | 5 | 6 | 5 | 3 | 3 | 4 | 2 | 1 | 1 | 2 | 4 |
| | % | 2·6 | 3 | 0·5 | 2 | 2 | 1 | 4 | 2·5 | 3 | 2 | 4 | 3 | 3·2 | 3·8 | 3·2 | 3·8 | 3·2 | 1·9 | 3 | 4 | 2·1 | 1 | 1·7 | 3·4 | 2 |
| Salivary gland | N | | 1 | | | | | | 1 | | | 1 | | | | | | | | | | 1 | | | | 1 |
| | % | | 0·5 | | | | | | 0·5 | | | 0·5 | | | | | | | | | | 1 | | | | 0·5 |
| Spleen | N | 1 | 1 | 2 | 2 | 2 | 1 | 2 | 2 | 3 | 2 | 1 | 1 | 1 | 2 | 1 | 4 | | 3 | 1 | | | 1 | | | 1 |
| | % | 0·5 | 0·5 | 1 | 1 | 1 | 0·5 | 1 | 1 | 1·5 | 1 | 0·5 | 0·5 | 0·6 | 1·2 | 0·6 | 2·5 | | 1·9 | 1 | | | 1 | | | 0·5 |
| Stomach—fore | N | 1 | 1 | | 1 | 1 | | 2 | | | | | | 1 | | | | 1 | 1 | | | | | | | |
| | % | 0·5 | 0·5 | | 0·5 | 0·5 | | 1 | | | | | | 0·6 | | | | 0·6 | 0·6 | | | | | | | |
| Stomach—glandular | N | | | | 1 | | | 1 | | | | | | | | | 1 | | | | | | | | | |
| | % | | | | 0·5 | | | 0·5 | | | | | | | | | 0·6 | | | | | | | | | |
| Thymus | N | 2 | 1 | 1 | 4 | 1 | | 1 | | | | | 3 | | 2 | 3 | 1 | 1 | | | | 1 | | 1 | | 5 |
| | % | 1 | 0·5 | 0·5 | 2 | 0·5 | | 0·5 | | | | | 1·5 | | 1·2 | 1·9 | 0·6 | 0·6 | | | | 1 | | 1·7 | | 2·5 |
| Thyroid | N | | | 1 | 1 | 1 | 1 | 2 | 1 | | | | 1 | | 2 | 2 | | | 1 | | | | | | | 2 |
| | % | | | 0·5 | 0·5 | 0·5 | 0·5 | 1 | 0·5 | | | | 0·6 | | 1·2 | 1·3 | | | 0·6 | | | 1 | | | | 1 |
| Total of tumour-bearing animals (excluding skin tumours) | N | 119 | 120 | 107 | 117 | 111 | 117 | 124 | 124 | 117 | 116 | 109 | 102 | 95 | 105 | 99 | 91 | 110 | 98 | 63 | 66 | 59 | 68 | 29 | 29 | 114 |
| | % | 61 | 60 | 53·8 | 59·1 | 55·8 | 58·5 | 62 | 62 | 58·5 | 58·3 | 54·5 | 51 | 60·1 | 65·6 | 62·7 | 56·9 | 71 | 61·6 | 63 | 66·7 | 60·8 | 68 | 48·3 | 49·2 | 57·8 |

* A size and B size combined.
F = Filter; P = Plain; N = Number.

### TABLE VIII.—*General Histopathology—Summary*

Treatment and dose (mg/wk)*

| | | 100% Tob | | | | | | 80% Tob/20% NSM | | | | | | 55% Tob/45% NSM | | | | | | 100% NSM | | | | | | |
|---|---|---|---|---|---|---|---|---|---|---|---|---|---|---|---|---|---|---|---|---|---|---|---|---|---|---|
| | | 75 | | 126 | | 210 | | 75 | | 126 | | 210 | | 75 | | 126 | | 210 | | 126 | | 210 | | 300 | | |
| | | F | P | F | P | F | P | F | P | F | P | F | P | F | P | F | P | F | P | F | P | F | P | F | P | Controls |
| Number of animals examined | | 194 | 200 | 199 | 192 | 199 | 200 | 200 | 200 | 200 | 199 | 200 | 200 | 158 | 160 | 158 | 160 | 155 | 159 | 100 | 99 | 97 | 100 | 60 | 59 | 198 |
| Generalized | N | 7 | 3 | 5 | 6 | 10 | 8 | 5 | 7 | 7 | 9 | 6 | 19 | 3 | 5 | 3 | 4 | 3 | 3 | 3 | 2 | 1 | 4 | 2 | 3 | 5 |
| amyloidosis | % | 3·5 | 1·5 | 2·5 | 3 | 5 | 4 | 2·5 | 3·5 | 3·5 | 4·5 | 3 | 9·5 | 2 | 3 | 2 | 2·5 | 2 | 2 | 3 | 2 | 1 | 4 | 3·5 | 5 | 2·5 |
| Bladder | N | 11 | 9 | 9 | 8 | 12 | 12 | 9 | 12 | 14 | 9 | 13 | 15 | 4 | 6 | 5 | 7 | 13 | 7 | 2 | 5 | 7 | 3 | 1 | 1 | 10 |
| cystitis | % | 5·5 | 4·5 | 4·5 | 4 | 6 | 6 | 4·5 | 6 | 7 | 4·5 | 6·5 | 7·5 | 2·5 | 4 | 3 | 4·5 | 8·5 | 4·5 | 2 | 5 | 7 | 3 | 1·5 | 1·5 | 5 |
| Liver, | N | 16 | 21 | 29 | 17 | 6 | 22 | 23 | 19 | 26 | 21 | 20 | 21 | 17 | 8 | 17 | 16 | 14 | 6 | 11 | 1 | 10 | 7 | 7 | 5 | 22 |
| Tyzzer's disease | % | 8 | 10·5 | 14·5 | 9 | 3 | 11 | 11·5 | 9·5 | 13 | 10·5 | 10 | 10·5 | 11 | 5 | 11 | 10 | 9 | 4 | 11 | 1 | 10·5 | 7 | 11·5 | 8·5 | 11 |
| Lung, chronic | N | 62 | 54 | 65 | 54 | 67 | 55 | 48 | 65 | 66 | 53 | 54 | 65 | 49 | 49 | 46 | 46 | 39 | 49 | 30 | 26 | 27 | 36 | 19 | 15 | 61 |
| pulm. disease | % | 31 | 27 | 32·5 | 28 | 33·5 | 27·5 | 24 | 32·5 | 33 | 26·5 | 27 | 32·5 | 31 | 30·5 | 29 | 29 | 25 | 31 | 30 | 26·5 | 28 | 36 | 31·5 | 25·5 | 31 |
| Ovary, cystic | N | 18 | 16 | 21 | 18 | 20 | 17 | 19 | 23 | 19 | 16 | 17 | 17 | 19 | 14 | 15 | 11 | 7 | 7 | 10 | 10 | 11 | 10 | 10 | 6 | 15 |
| changes | % | 9 | 6 | 10·5 | 9·5 | 10 | 8·5 | 9·5 | 11·5 | 9·5 | 8 | 8·5 | 8·5 | 12 | 9 | 9·5 | 7 | 4·5 | 4·5 | 10 | 10 | 11·5 | 10 | 16·5 | 10 | 7·5 |
| Cervix and uterus, | N | 16 | 29 | 17 | 19 | 14 | 16 | 16 | 23 | 25 | 21 | 23 | 30 | 23 | 15 | 18 | 26 | 14 | 14 | 8 | 13 | 8 | 11 | 10 | 6 | 27 |
| hydrometra and pyometra | % | 8 | 14·5 | 8·5 | 10 | 7 | 8 | 8 | 11·5 | 12·5 | 10·5 | 11·5 | 15 | 14·5 | 9·5 | 11·5 | 16·5 | 9 | 9 | 8 | 13 | 8 | 11 | 16·5 | 10 | 13·5 |
| Haemorrhage/ | N | 5 | 7 | 9 | 6 | 5 | 7 | 2 | 7 | 4 | 1 | 5 | 8 | 6 | 4 | 2 | 7 | 4 | 8 | 2 | 5 | 3 | 5 | 1 | 3 | 4 |
| thrombus | % | 2·5 | 3·5 | 4·5 | 3 | 2·5 | 3·5 | 1 | 3·5 | 2 | 0·5 | 2·5 | 4 | 4 | 2·5 | 1 | 4·5 | 2·5 | 5 | 2 | 5 | 3 | 5 | 1·5 | 5 | 2 |
| Endometrial | N | 35 | 29 | 32 | 31 | 27 | 25 | 23 | 35 | 29 | 24 | 25 | 26 | 26 | 21 | 17 | 23 | 22 | 29 | 15 | 23 | 15 | 15 | 8 | 2 | 30 |
| hyperplasia/cysts | % | 18 | 14·5 | 16 | 16 | 13·5 | 12·5 | 11·5 | 17·5 | 14·5 | 12 | 12·5 | 12·5 | 16·5 | 13 | 11 | 14·5 | 14 | 18 | 15 | 23 | 15·5 | 15 | 13·5 | 3·5 | 15 |
| Heart | N | 2 | 4 | 5 | 1 | 2 | 2 | 4 | 6 | 3 | 4 | 4 | 4 | 3 | 2 | 2 | 2 | 4 | 2 | 2 | 1 | 1 | 2 | 0 | 2 | 4 |
| myocarditis | % | 1 | 2 | 2·5 | 0·5 | 1 | 1 | 2 | 3 | 1·5 | 2 | 2 | 2 | 2 | 1 | 1·5 | 1·5 | 2·5 | 1·5 | 2 | 1 | 1 | 2 | 0 | 3·5 | 2 |
| Kidney, | N | 33 | 23 | 38 | 29 | 50 | 36 | 39 | 30 | 38 | 36 | 30 | 28 | 20 | 21 | 15 | 15 | 28 | 24 | 19 | 14 | 9 | 25 | 10 | 6 | 33 |
| nephropathy | % | 17 | 11·5 | 19 | 15 | 25 | 18 | 19·5 | 15 | 19 | 18 | 15 | 14 | 12·5 | 13 | 9·5 | 9·5 | 18 | 15 | 19 | 14 | 9·5 | 25 | 16·5 | 10 | 16·5 |

* A size and B size combined.
F = Filter; P= =Plain; N = Number.

TABLE IX.—*The Overall Incidence of Tumours and Lymphomas, Excluding Skin Tumours*

| | Dosage | | | | | | | |
|---|---|---|---|---|---|---|---|---|
| | 75 | | 126 | | 210 | | 300 | |
| Treatment | Lymphoma | Other tumours | Lymphoma | Other tumours | Lymphoma | Other tumours | Lymphoma | Other tumours |
| 100% Tob. | 23 | 60·5 | 20 | 55 | 19 | 57 | | |
| 80% Tob./ 20% NSM | 21 | 62 | 22 | 58 | 17 | 53 | | |
| 55% Tob./ 45% NSM | 25 | 63 | 20 | 60 | 22 | 66·5 | | |
| 100% NSM | | | 24·5 | 64·5 | 21 | 63·5 | 14 | 48 |
| Control | Lymphomas 24; Other tumours 58 | | | | | | | |

ments to test this possibility (to be published) show little or no significant effect of glycerol on skin-tumour incidence in mice when it is added to tobacco condensates before skin application.

Similarly, it is known that acetone can react with glycerol, in the presence of acid, to form solketal (isopropylidene glycerol—Renoll and Newman, 1968). If this occurred to any great extent, the condensate doses would be incorrectly calculated, and again there could be some interference with the interaction between the carcinogenic moiety and the mouse skin. Analysis has shown that less than 1·5% solketal was formed in the condensates used in this experiment, and that, even under the most favourable conditions, only 3% was formed after 7 weeks' storage. Such small amounts could not result in the marked reduction of activity found in this study. The possibility of phase separation of condensate before application, to which substitute condensates are sometimes prone, was avoided in this experiment by shaking the solution before application. Without this, it is obviously possible to apply different phases which might possess different activities (Chortyk and Bock, 1976).

The small yield of NSM condensate results in a blend condensate which is preponderantly of tobacco origin. The yield of condensate from an 80% tobacco blend is theoretically 85% that of a normal cigarette, but of this condensate 94% could be attributable to tobacco. Even at 55% tobacco, 83% of the content of the much-reduced blend condensate is derived from tobacco. It was this relatively minor variation in the effective tobacco contribution in the various blend condensates which made necessary the very large numbers of mice in the trial, if valid statistical conclusions were to be drawn. A major source of concern was the possibility that any reduction in activity achieved by adding NSM to a blend could be offset by an adverse interaction caused by the conjoint burning of NSM and tobacco. No such interaction is demonstrated.

In addition, these minor changes could be influenced in a major way by slight variation in condensate yields during the smoking of the different cigarette types. It is probably more realistic to compare the relative activities of the different blends on the basis of the cigarettes smoked to produce the doses of condensate. This has the added advantage of giving the combined effect of reduced condensate and reduced condensate activity.

Thus it may be calculated that a combination of filter and 20% NSM results in about 33% less of fresh anhydrous smoke condensate per cigarette, than with the unfiltered 100% tobacco cigarette (Table I), and that the condensate itself shows less tumour-producing activity than that derived from tobacco (Table V). The combined effect could result in a cigarette which shows over 40% less activity than the unfiltered tobacco

TABLE X.—*Comparisons of the Effect of NSM and Filters on the FAS Condensate Yield and Skin Tumour Effects* (%)

| Cigarette type | Treatment | Overall tumour rate | Relative* activity of condensate | Number of B plain 100% Tob. cigarettes equivalent to 100 test cigarettes |
|---|---|---|---|---|
| | 100% Tob. | 25·8 | 100 | 100 |
| B plain | 80% Tob./20% NSM | 24·1 | 95·5 | 85·7 |
| | 55% Tob./45% NSM | 18·5 | 81·2 | 59·1 |
| | 100% Tob. | 24·6 | 95·2 | 71·2 |
| B filter | 80% Tob./20% NSM | 17·6 | 80·3 | 56·0 |
| | 55% Tob./45% NSM | 13·3 | 71·8 | 43·2 |
| | 100% Tob. | 18·1 | 82·1 | 73·9 |
| A plain | 80% Tob/.20% NSM | 17·9 | 79·6 | 71·9 |
| | 55% Tob./45% NSM | 14·6 | 71·5 | 51·2 |
| | 100% Tob. | 20·0 | 88·2 | 55·9 |
| A filter | 80% Tob./20% NSM | 18·7 | 85·6 | 49·7 |
| | 55% Tob./45% NSM | 9·4 | 63·3 | 29·1 |

* Derived from regression analyses on values of Weilbull *b*.

cigarette (Table X). These figures relate to the control tobacco used in these experiments, which yields greater amounts of condensate than are commonly produced from the commercial blends of today. Similar calculations for different types (Table X) show the relative effects of combining filter and substitute.

Skin hyperplasia (in the absence of other skin tumours) was closely linked to the amount of tobacco condensate painted on the mouse, showing a strong dose–response relationship. There were indications that, as the proportion of NSM increased in the blend, there was a slight reduction of hyperplasia; 100% NSM condensate producing virtually no hyperplasia. These results would suggest that NSM makes little qualitative contribution to those factors responsible for hyperplasia.

There was a possibility that a proportion of the applied condensate was absorbed through the skin or ingested while grooming, and that this might lead to systemic pathology. The absence of any systemic pathology related to a particular treatment shows that there is no constituent of the condensate which is likely to result in marked and unsuspected pathology, if absorption occurs.

The relevance of the mouse skin model to human carcinogenesis remains uncertain. If it is regarded as a model system to determine the comparative effects of smoke condensates on a tissue in terms of neoplastic response, then the present work indicates that NSM condensate is significantly less carcinogenic than tobacco condensate, and offers a method compatible with others to reduce the potential hazards of cigarette smoking.

The incorporation of NSM into tobacco cigarettes would have an effect comparable to a reduction in the number of tobacco cigarettes smoked. If there is a linear relationship between numbers smoked and lung cancer (Doll and Pike, 1972) and if the mouse skin model applies, then the expected reductions in lung cancer incidences would approximate the amount of NSM present. Confirmation of this will require prolonged inhalation studies in experimental animals capable of responding to inhaled smoke by the development of bronchopulmonary tumours, or by prolonged exposure in man with the attendant epidemiological studies. In the absence of precise understanding of the carcinogenic mechanisms operating in mouse skin, however, it still has validity only as a model for comparing the relative potencies of topically applied materials.

It is worth noting that the relevance of these comments would be severely reduced if the activity of NSM condensate was not less than that of the control material derived from tobacco. If this was not so, it would be difficult to predict with confidence a beneficial effect to the human smoker, even if the amount of condensate was reduced. Given the variability of dosage which actually occurs over the long periods of exposure of the human smoker, due for example to depth of inhalation or rate of smoking, mere reduction of condensate yield might be more than offset by increased potency, or the increased sensitivity of the human bronchus compared with mouse skin. The safest course must be to reduce both the amount and activity of the tar.

We acknowledge with gratitude the invaluable help provided by Mr J. V. Gregg and Mr W. S. Paige in the experimental design and statistical analyses.

### REFERENCES

BRAVEN, J., BONKER, G. J., FENNER, M. L. & TONGE, B. L. (1967) The Mechanism of Carcinogenesis by Tobacco Smoke. Some Experimental Observations and a Hypothesis. *Br. J. Cancer*, **21**, 623.

CHORTYK, T. & BOCK, F. G. (1976) Tumour-promoting Activity of Certain Extracts of Tobacco. *J. natn. Cancer Inst.*, **56**, 1041.

DAY, T. D. (1967) Carcinogenic Action of Cigarette Smoke Condensate on Mouse Skin. An Attempt at a Quantitative Study. *Br. J. Cancer*, **21**, 56.

DOLL, R. & PIKE, M. C. (1972) Trends in Mortality among British Doctors in Relation to their Smoking Habits. *J. R. Coll. Phys.*, **6**, 216.

HUNTER, B. (1971) Eradication of Tyzzer's Disease in a Colony of Barrier-maintained Mice. *Lab. Animals*, **5**, 271.

LEUCHTENBERGER, C. & LEUCHTENBERGER, R. (1969) Cytologic and Cytochemical Effects on Primary Mouse Kidney Tissue and Lung Organ Cultures after Exposure to Whole, Fresh Smoke and its Gas Phase from Unfiltered, Charcoal-filtered, and Cigar Tobacco Cigarettes. *Cancer Res.*, **29**, 862.

LEUCHTENBERGER, C., LEUCHTENBERGER, R. & HORISBERGER, M. (1967) Change of Frequency and of Spectrum of Tumours in Snell's Mice after Chronic Inhalation of Fresh Intermittent Cigarette Smoke. *Proc. Am. Ass. Cancer Res.*, **8**, 40.

PETO, R. & LEE, P. N. (1973) Weibull Distribution for Continuous–carcinogenic–experiments. *Biometrics*, **29**, 457.

PIKE, M. C. (1966) A Method of Analysis of a Certain Class of Experiments in Carcinogenesis. *Biometrics*, **22**, 142.

RENOLL, M. & NEWMAN, M. S. (1968) Isopropylidene glycerol. *Organic Syntheses Collective Vol.*, **3**, 502.

ROTHWELL, K. & GRANT, C. A. (1974) Standard Methods for the Analysis of Tobacco Smoke. Tobacco Research Council, Research Paper 11, 2nd edn, London.

SAUNDERS, L. Z. (1958) Tyzzer's Disease. *J. natn. Cancer Inst.*, **20**, 893.

VAN DUUREN, B. L. (1968) Tobacco Carcinogenesis. *Cancer Res.*, **28**, 2357.

WYNDER, E. L. & HOFFMANN, D. (1968) Experimental Tobacco Carcinogenesis. *Science, N.Y.*, **162**, 862.

YULE, G. U. (1934) On Some Points Relating to Vital Statistics, more especially Statistics of Occupational Mortality. *J. R. Stat. Soc.*, **97**, 1.